# Effects of Cement Shade, Cementation, and Thermocycling on the Color Parameters and the Final Color of the SLA-Printed Photopolymer Resins †

**DOI:** 10.3390/polym17233127

**Published:** 2025-11-25

**Authors:** Esra Kaynak Öztürk, Elif Yılmaz Biçer, Beyza Güney, Sina Saygılı, Nagehan Aktaş, Merve Bankoğlu Güngör

**Affiliations:** 1 Department of Prosthodontics, Faculty of Dentistry, Gazi University, Ankara 06490, Türkiye; esrakaynak@gazi.edu.tr (E.K.Ö.); elifyilmaz1@gazi.edu.tr (E.Y.B.); beyzabayat@gazi.edu.tr (B.G.); 2 Department of Prosthodontics, Faculty of Dentistry, İstanbul University, İstanbul 34116, Türkiye; sinasaygili@istanbul.edu.tr; 3 Department of Pediatric Dentistry, Faculty of Dentistry, Gazi University, Ankara 06490, Türkiye; nagehanaktas@gazi.edu.tr

**Keywords:** 3D printing, color difference, color parameters, photopolymer resins, SLA

## Abstract

This study investigated the effects of resin cement shade and thermocycling on the color parameters and final appearance of SLA-printed photopolymer resins. Specimens with a thickness of 1 mm were fabricated and categorized into eight groups based on four different cement shades (universal-A2, clear, white, and opaque) and two applications (cementation and thermal aging). Differences in color parameters (ΔL*, Δa*, and Δb*) were measured after cement polymerization and thermocycling, and overall color differences (ΔE_00_-1 and ΔE_00_-2) were calculated. Two-way ANOVA revealed significant interactions between cement shade and thermocycling for ΔL*, Δa*, and Δb* (*p* < 0.05). After cementation, L* decreased for universal-A2, clear, and white cements, but increased for opaque cement. Furthermore, thermocycling altered L*, a*, and b* values differently among the experimental groups. Cement shade significantly influenced ΔE_00_, with universal-A2 and clear cements showing higher values than white and opaque cements (*p* < 0.05). All ΔE_00_ values exceeded the clinically acceptable limit (>1.8). The findings suggest that careful selection of the cement shade is therefore critical to achieving optimal esthetic outcomes with the tested 3D-printed resin. Formlabs SLA-printed permanent resin, although labeled as an A2 shade, behaves more like white or opaque shades, highlighting inconsistencies between labeled and actual color.

## 1. Introduction

In the past, digital dentistry predominantly relied on subtractive manufacturing (SM) methods, in which dental prostheses were produced by milling prefabricated blocks or disks through computer-aided design and computer-aided manufacturing (CAD-CAM) systems. While this method offered precision and consistency, it also resulted in considerable material waste and limitations in design complexity. More recently, additive manufacturing (AM) or three-dimensional (3D) printing has gained increasing prominence in this field. The 3D printing technology constructs dental structures layer by layer from digital models, enabling greater design freedom, material efficiency, and customization [1,2]. Furthermore, improvements in additive resin materials have facilitated the transition from subtractive manufacturing to additive manufacturing. The use of these materials has enabled the digital fabrication of removable prostheses, crowns, and fixed partial dentures with reduced time and cost while allowing complex designs. However, its full potential depends on further advances in materials and production processes [1,3], and studies are ongoing to evaluate the mechanical properties [4,5], dimensional accuracy [6], fit [7,8], biocompatibility [9], and esthetic characteristics [10,11] of 3D-printed resins developed for long-term restorative purposes. Although 3D-printed permanent resin restorations are clinically accepted, they are relatively new and are considered a less common choice for indirect restorations [10].

Several factors influence the optical properties of 3D-printed permanent resins. The composition of the resin material, its filler content, and matrix structure directly affect optical characteristics. In addition, 3D printing technology, including printing resolution, layer thickness, post-processing steps, and surface finishing procedures, also plays a significant role in shaping optical outcomes [12,13,14]. There are various production technologies in additive manufacturing, including stereolithography (SLA), digital light processing (DLP), and liquid crystal display (LCD), each with its own advantages and disadvantages [15]. SLA printers use a laser beam that moves across the bottom of a resin vat to trigger the polymerization process. In contrast, DLP and LCD printers project an entire layer image at once onto the bottom surface of the vat. Polymerization occurs wherever the resin is exposed to ultraviolet or multi-wavelength light in DLP systems, or to LED light passing through an LCD panel in LCD systems [16]. Furthermore, a wide range of materials is available for fabricating restorations, each offering distinct properties and clinical applications [1]. In the present study, the material of choice was Permanent Crown Resin, produced by Formlabs, which is one of the few materials currently available for fabricating 3D-printed permanent crowns. This material is a type of hybrid material containing approximately 30% to 50% glass fillers by weight [17]. Although it exhibited surface roughness similar to that of milled hybrid composite materials, its microhardness was found to be comparatively lower [1,5]. Variations in production-related parameters can impact the esthetic harmony of the restoration with natural teeth and overall patient satisfaction [14,18,19]. Additionally, understanding the changes in optical properties of 3D-printed resins over time is critical for optimizing dental restorative materials and achieving restorations that are both durable and clinically consistent [11]. Many other factors influence the final color and color stability of restorations. Among these, cement-related factors may have a significant impact. Resin cements, which include bonding agents, primers, and various colors, are commonly used for cementing fixed restorations. The availability of multiple color options is essential for achieving optimal esthetic outcomes in dental restorations [20]. Previous studies have reported that the color of luting cement has a negligible impact when both the tooth substrate and restoration thickness are within favorable limits [21,22,23]. Under these conditions, the natural translucency and masking ability of the restorative material are typically sufficient to ensure an esthetically pleasing outcome, with minimal visual interference from the cement layer. However, in clinical scenarios where the restoration thickness is less than 1.5 mm or when it is applied over a discolored tooth or a dark underlying abutment, the color of the luting cement becomes significantly more critical. In such cases, the translucency of the restorative material may allow the underlying substrate and cement shade to influence the final appearance, potentially compromising esthetic results. Therefore, selecting appropriately shaded cement may help to mask underlying discolorations [21,24,25,26]. In addition to cement-related factors, the color stability of restorations can also be affected over time due to intraoral conditions. To simulate these effects in laboratory settings, various artificial aging protocols are employed. Among these, thermocycling is a widely used method that mimics the thermal stresses to which restorations are exposed in the oral environment.

While the optical properties and color stability of subtractively manufactured CAD-CAM materials have been extensively investigated, there remains limited evidence regarding esthetic outcomes of additively manufactured permanent crown resins, particularly those fabricated using stereolithography (SLA) technology. Existing studies on 3D-printed dental materials have predominantly focused on denture base resins or provisional materials [27,28,29]. Moreover, although cement shade and thermocycling are both known to affect the final color of resin-based restorations, no previous study has comprehensively evaluated their combined impact on the color parameters and color difference of SLA-printed permanent crown resins. Therefore, this study aimed to assess the effects of both cement shade and thermocycling on the color parameters and final color outcome of SLA-printed photopolymer resin restorations. The null hypothesis of the study was that the cement shade and the cementation/thermocycling would not have a statistically significant effect on the color parameters or the final color of the SLA-printed photopolymer resins.

## 2. Materials and Methods

### 2.1. Sample Size Calculation and Study Design

**Figure 1:**
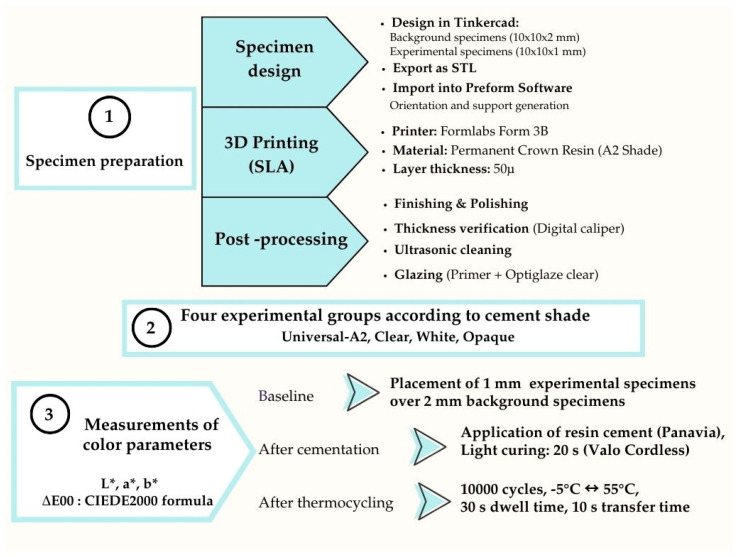
Flowchart of the study design.

A power analysis was performed using G*Power software (version 3.1.9.4) to calculate the minimum required sample size for each experimental group. It was decided to analyze the difference in color parameters and color differences data using a two-way ANOVA in which the cement shade and application were the independent factors. Using a significance level of α = 0.05, a statistical power of 0.80, and an effect size (*f*) of 0.40, the total required sample size was determined to be 73, corresponding to a minimum of approximately 10 specimens per group. Four experimental groups were generated based on the cement shades (universal-A2, clear, white, and opaque), and two groups were generated based on the application (cementation and thermocycling). The flowchart is summarized in Figure 1.

### 2.2. Specimen Preparation

Background specimens were designed in a square shape (10 mm × 10 mm) with a uniform thickness of 2.0 mm, using TinkerCAD software (Autodesk Inc., San Rafael, CA, USA). These background specimens represent the underlying tooth structure. The experimental specimens were designed to match the dimensions of the background specimens (10 mm × 10 mm), while maintaining a standardized thickness of 1.0 mm. All digital designs were exported in standard tessellation language (STL) format and subsequently imported into PreForm software (Formlabs Inc., Somerville, MA, USA) to optimize orientation and generate support structures before the fabrication process.

**Figure 2:**
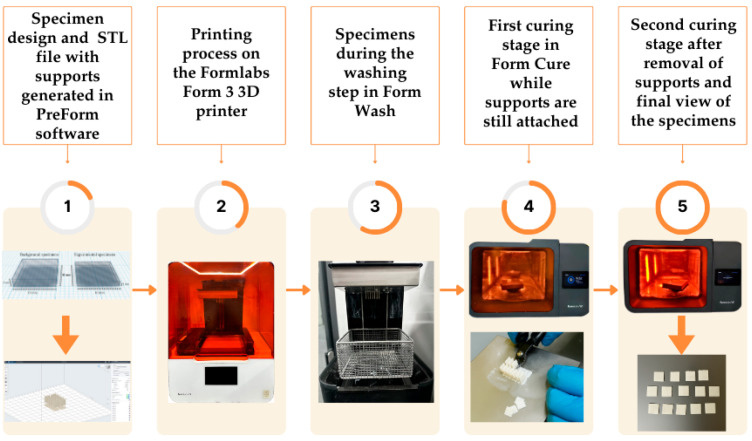
Production steps of the SLA-printed specimens.

Specimens were printed with Permanent Crown Resin (Formlabs, Somerville, MA, USA) in A2 shade, ceramic-filled resin for 3D printing of permanent dental restorations using a 3D printer (Formlabs Form 3B 3D printer, Formlabs, Somerville, MA, USA). The SLA printing process was carried out according to the manufacturer’s recommended parameters, employing a layer thickness of 50 µm and the exposure time automatically defined by the software based on the selected resin. Detailed information on the permanent crown resin is presented in Table 1. An overview of the fabrication and post-processing steps of the SLA-printed resin specimens is presented in Figure 2.

The specimens were finished and polished using polishing rubber point sets (CPD 14 Composite Hybrid Ceramic 3D Printing Hybrid Material; G&Z Instrumente GmbH, Munich, Germany). The thickness of each specimen was verified using a digital caliper. All specimens were cleaned in distilled water using an ultrasonic cleaner. Glazing was performed on the specimens. A single surface of each glazed specimen was first coated with primer (GC Corporation, Tokyo, Japan), followed by the application of glaze material (Optiglaze Clear; GC Corporation, Tokyo, Japan) in clear shade. The specimens were then polymerized for 40 s using a polymerization unit (Valo Cordless; Ultradent Products Inc., South Jordan, UT, USA).

The specimens were cleaned in an ultrasonic cleaner for 3 min and air-dried. Four groups were prepared based on the resin cement shade: universal-A2, clear, white, and opaque (Panavia V5; Kuraray Noritake Dental Inc., Tokyo, Japan). The resin cement was dispensed using an automix syringe equipped with a mixing tip. Before application, a small amount of cement was extruded and discarded to ensure homogeneous mixing. A uniform, thin coating of cement was placed on the cementation surface of the 2 mm-thick background specimens. The 1 mm-thick experimental specimen was then positioned on the background specimen using finger pressure to achieve a consistent cement thickness. Excess cement was removed. Polymerization was performed using the same polymerization unit for 20 s, as recommended by the manufacturer.

### 2.3. Thermocycling Procedure

Thermocycling was performed using a thermocycling device (MTE 101; MOD Dental, Esetron Robotechnologies, Ankara, Türkiye). All specimens underwent 10,000 thermal cycles between water baths maintained at 5 °C and 55 °C, with a dwell time of 30 s in each bath and a 10 s transfer time between them. All specimens underwent 10,000 cycles, which simulated one year of clinical use [30].

### 2.4. Measurements of the Color Parameters

**Figure 3:**
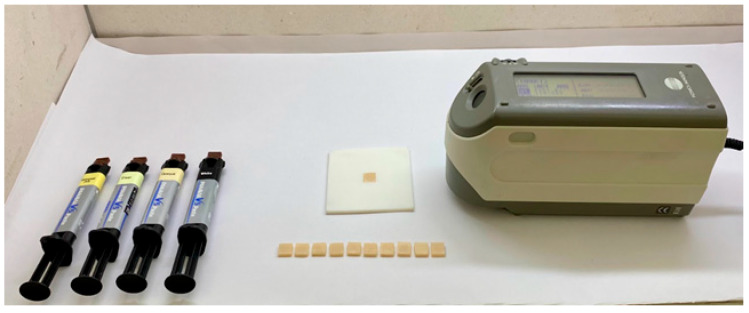
Measurement of the color parameters.

Measurements of the color parameters were performed at three stages: (1) after specimen preparation (baseline), (2) after cement polymerization, and (3) after thermocycling. A spectrophotometer (Konica Minolta 2300d; Konica Minolta Inc., Tokyo, Japan) was used under standardized conditions: 6500 K daylight illuminant, diffuse/8° geometry, 10° standard observer angle, specular component excluded (SCE) mode, and an 8 mm diameter measurement aperture. Color parameters were taken three times for each specimen. Before the measurements of all groups, the spectrophotometer was calibrated. To ensure the consistent positioning of all specimens during color measurements, a white 3D-printed mold was used, and each specimen was placed within this mold during the measurements (Figure 3).

The L* value denotes the lightness of a color, ranging from 0 (black) to 100 (white). The a* coordinate represents color variation along the red-green axis, with positive values indicating a shift toward red and negative values indicating a shift toward green. The b* coordinate reflects color variation along the yellow–blue axis, where positive values indicate a shift toward yellow and negative values indicate a shift toward blue [31]. Two ΔL*, Δa*, and Δb* values were calculated for each specimen. ΔL*_1_ and ΔL*_2_ stated the difference in L* value between cement polymerization-baseline and after thermocycling-cement polymerization. Δa*_1_ and Δa*_2_ indicated the difference in a* value between cement polymerization-baseline and after thermocycling-cement polymerization. Δb*_1_ and Δb*_2_ stated the difference in b* value between cement polymerization-baseline and after thermocycling-cement polymerization.

Two color difference values (ΔE_00_-1: baseline to after cement polymerization and ΔE_00_-2: after cement polymerization to after thermocycling) were calculated using the CIEDE2000 formula [11]. These values were then compared against established perceptibility and acceptability thresholds. According to Paravina et al. [32], the perceptibility threshold for ΔE_00_ is 0.8, meaning that color differences below this value are generally imperceptible to the human eye. The acceptability threshold is 1.8, indicating the maximum color difference considered clinically acceptable by most observers. Values exceeding 1.8 suggest a noticeable and potentially unacceptable color mismatch, while values between 0.8 and 1.8 may be perceptible but still considered acceptable under clinical conditions [32].

### 2.5. Statistical Analyses

The color difference data were analyzed using statistical software (IBM SPSS Statistics for Windows, v20.0; IBM Corp., Armonk, NY, USA). The normality of the data distribution was assessed using the Shapiro–Wilk test. The data were normally distributed; thus, a two-way analysis of variance (ANOVA) was performed to evaluate the effects of the cement shade and application on the color parameters and color difference values. The homogeneity of the variances was tested with the Levene test. The Tukey HSD test was used for post hoc pairwise comparisons. Results were considered statistically significant when *p* < 0.05.

## 3. Results

The results of the two-way ANOVA for the ΔL*, Δa*, and Δb* values revealed an interaction between the cement shade and application factors (*p* < 0.05). The mean values, standard deviations, and comparative results of the ΔL* values are presented in Table 2. The L* values for universal-A2, clear, and white cement shades decreased after cementation, while the L* value of the opaque cement increased after cementation. The ΔL* values for white and opaque cements were significantly different from those of the other cement shade groups; however, there was no statistical difference between the ΔL* values of universal-A2 and clear cement shades (*p* = 0.436; *p* > 0.05). The L* values of universal-A2 and clear cement shades decreased, and the L* values of white and opaque cement shades increased after thermocycling. The ΔL* values of the white and opaque cements were significantly higher than the ΔL* values of the universal-A2 and clear cement groups (*p* < 0.05). The ΔL* values were significantly different between the cementation and thermocycling groups for all cement shades (*p* < 0.05) except for the opaque shade cement (*p* = 0.771; *p* > 0.05).

The mean values, standard deviations, and comparative results of the Δa* values are presented in Table 3. The a* values after cementation increased in the universal-A2 and clear cements, and the Δa* values were not significantly different between these groups (*p* = 0.909). However, the a* values decreased in the white and opaque cements. The results demonstrated that thermocycling caused significant differences in the Δa* values of all cement shade groups. The Δa* values were significantly lower in the universal-A2 and clear cements (*p* < 0.05); however, the Δa* values were higher in the white and opaque cements (*p* < 0.05). The mean values, standard deviations, and comparative results of the Δb* values are presented in Table 4. The b* values after cementation decreased for the universal-A2, clear, and white cement shades, while the b* value increased after cementation for the opaque cement shade. Δb* value for the opaque cement shade was significantly higher than the other cement shade groups (*p* < 0.05). Thermocycling significantly decreased the b* values of all cement shade groups (*p* < 0.05).

The results of the two-way ANOVA for the ΔE_00_ values showed that there was no interaction between the cement shade and application factors (*p* = 0.158, *p* > 0.05). The application factor (cementation and thermocycling) did not have a significant effect on the ΔE_00_ values (*p* = 0.216, *p* > 0.05). However, cement shade significantly affected the ΔE_00_ values (*p* < 0.05). The mean values, standard deviations, and comparative results of the ΔE_00_ values are presented in Table 5. The ΔE_00_ values of the universal-A2 and clear cements were significantly higher than those of the white and opaque cement shade groups (*p* < 0.05). When compared with perceptibility (0.8) and acceptability (1.8) threshold values, the results indicated that the ΔE_00_ values in the experimental groups exceeded the clinically acceptable limit (>1.8). Additionally, although cementation and thermocycling did not have a statistically significant effect on the color difference, they caused a clinically unacceptable color difference in SLA-printed specimens with a thickness of 1 mm.

## 4. Discussion

The effects of the cement shade (universal-A2, clear, white, and opaque) and the application (cementation and thermocycling) on the color parameters and the final color of SLA-printed photopolymer resins were investigated. The two-way ANOVA results showed that the cement shade and the application were effective on the ΔL*, Δa*, and Δb* values (*p* < 0.05); however, only the cement shade was effective on the ΔE_00_ values. Thus, the null hypothesis of the study that neither the cement shade nor the application would have a statistically significant effect on the color parameters and the final color of the SLA-printed resins was partially rejected. However, although no statistically significant differences were found in the color difference values among the experimental groups, it is worth noting that visually perceptible color differences were observed in all groups after cementation and thermocycling.

Today, numerous permanent resins and related manufacturing techniques are available in the dental market. Although the mechanical and optical advantages of current printable resin materials are frequently investigated, varying results exist on this issue. In the present study, a permanent resin produced by Formlabs using the SLA technique was used. Stefaniak et al. [16] reported significant differences in particle and chemical emissions depending on the brand of SLA printer and resin color, indicating that printer configuration may affect the final characteristics of the material. Even among printers using the same resin type, variations in hardware design, such as resin heaters, can significantly influence the polymerization behavior and surface chemistry, ultimately impacting the optical properties of the printed object [16]. The surface finishing procedures applied to the material surface also influence the changes in the material properties that occur due to aging in the oral environment [33,34]. Albrecht et al. [33] evaluated the effect of aging on three 3D-printed resins (HarzLabs Dental Sand Pro, BEGO VarseoSmile Crown plus, Voco V-Print c&b temp) and one milled resin matrix ceramic (Voco Grandio Blocs) after polishing, glazing, or no treatment. Following 5000 thermal cycles, polishing and glazing reduced surface roughness; however, all groups exhibited significant color differences. Glazing caused greater shifts than polishing, though color stability remained clinically acceptable for all materials. In another study, Çakmak et al. [35] examined the effects of polishing methods and coffee thermocycling on additively manufactured (Crowntec and VarseoSmile) and subtractively manufactured (Cerasmart) resin-containing restoratives. Conventional polishing produced the smoothest surfaces and best color stability, whereas glazing (especially Vita Akzent LC) resulted in higher roughness and discoloration, particularly in Cerasmart. Coffee cycling had a minimal effect on the roughness but influenced the color, depending on the material and the polishing process. Studies have shown that both mechanical polishing and glazing are suitable surface finishing procedures for 3D-printed resin restorations, as both techniques contribute to improved surface roughness and optical properties [33,36,37,38]. In addition, Almejrad et al. [39] reported that 3D-printed interim restorations were highly prone to discoloration after immersing in wine, and glazing improved color stability compared to polishing. Accordingly, all specimens in the present study were glazed, as recommended by the manufacturer.

The CIEDE2000 color difference formula is currently considered one of the most reliable tools for evaluating color matching in dentistry. It provides results that more closely reflect human visual perception, making it especially useful in the color assessment of teeth and restorative materials. The formula defines perceptibility and clinical acceptability thresholds at 0.8 and 1.8, respectively, which has led to its growing use in analyzing tooth shade. Despite its relatively complex mathematical structure, the integration of digital technologies and software has enabled its efficient use in clinical practice [40,41]. In this study, color differences were determined using the CIEDE2000 formula, and the obtained values were evaluated against the perceptibility and clinical acceptability thresholds of 0.8 and 1.8, respectively.

In the present study, the differences in L*, a*, and b* values were evaluated after cementation with different shaded cements and subsequent thermocycling. Particularly when assessing the color parameters of ceramic or composite materials, the change in L* or ΔL* is a key parameter that reflects whether a material appears lighter or darker when compared to the natural tooth structure or a reference shade. In the CIELAB color space, the L* value denotes the lightness component of a color, with values ranging from 0, representing absolute black, to 100, representing pure white. A positive ΔL* indicates increased lightness, whereas a negative ΔL* reflects a darker appearance. Even slight variations in L* can influence esthetic outcomes, as human perception is susceptible to changes in brightness. Controlling the L* value through material selection, layering techniques, and surface finishing is essential for achieving harmonious shade integration in esthetic zones [42]. L* value is also regarded as more clinically relevant because variations in it are more perceivable [39]. The results of two-way ANOVA showed that ΔL* values were affected by the cement shade and application factors (*p* < 0.05). The results showed that the L* values, except for the opaque cement group, decreased in the universal-A2, clear, and white cement shade groups after cementation. The findings demonstrated that for the tested SLA-printed permanent resin with a thickness of 1 mm, the choice of shade of the luting cement significantly influenced the final shade of the restoration. Specifically, when universal-A2, clear, or white cements were applied, the restorations tended to exhibit a visibly darker appearance after cementation. In contrast, the use of opaque cement resulted in a lighter shade. These results can be attributed to the differences in the optical properties and pigment composition of the resin cements. The universal-A2 and clear cements, which have higher translucency and lower opacity, allow for greater light transmission and reflection from the underlying printed resin material. In contrast, white and opaque cements contain more opacifying fillers and brighter pigments, which initially mask the underlying substrate color and reflect more light. When examining the changes in L* values after thermocycling, it was observed that thermocycling caused less variation in L* values compared to cementation. After thermocycling, L* values decreased in restorations cemented with universal-A2 and clear cements, while L* values increased in those cemented with white and opaque cements. This indicated that thermocycling caused restorations cemented with A2-universal and clear cements to appear darker, whereas it caused those cemented with white and opaque cements to appear lighter. During thermocycling, water absorption and temperature fluctuations may alter the refractive index of both the cement and the resin matrix, leading to a reduction in brightness (L*), which can explain the darker appearance [43]. The L* value was evaluated against the 2.0 acceptability threshold; the AT for L* was established as −2 to +2 [39]. In the present study, the differences in L* values were higher than the AT after cementation with universal-A2, clear, and white cements. In comparison, the results were opposite for the opaque cement group after cementation and all thermocycling groups.

The a* coordinate in the CIELAB color space represents the chromatic axis, which ranges from green to red. A difference in a* (Δa*) between two color specimens reflects a shift along this red-green spectrum. Small changes in Δa* can affect the overall perception of color, particularly under natural lighting conditions. A positive Δa* indicates a shift toward a redder hue, while a negative value implies a greener appearance compared to the reference. Although the human eye is generally less sensitive to changes in a* than to lightness (L*), deviations in this coordinate can still influence the color harmony of dental restorations, particularly in the gingival or cervical regions where warmer tones are more prominent [44,45]. The results of two-way ANOVA showed that Δa* values were affected by the cement shade and application factors (*p* < 0.05). After cementation, the a* values increased in universal-A2 and clear cement shade groups. These results indicated that universal-A2 and clear cement shade groups had a higher red color content in the specimens. However, in the white and opaque cement groups after cementation, as well as in all cement shade groups after thermocycling, the a* values decreased. The results suggested that the red hue in the specimens diminished, while the green hue increased. The increase in a* values in the universal-A2 and clear cement groups can be attributed to their higher translucency, which allows more light to transmit through the resin substrate and enhances the red hue. In contrast, white and opaque cements contain more opacifying fillers and brighter pigments, which mask the substrate and reduce a* values by shifting the chroma toward green [46,47]. After thermocycling, the decrease in a* values across all groups is likely due to water absorption, hydrolytic degradation, and changes in the optical properties of both the resin matrix and cement, which increase light scattering and reduce the red component of the color [46,47].

The b* coordinate in the CIELAB color system represents the blue-yellow chromatic axis, where positive values indicate a shift toward yellow and negative values denote a shift toward blue. A difference in b* (Δb*) between two color specimens signifies a change in this dimension, which is particularly important in dental color matching, as natural teeth typically exhibit varying degrees of yellowish hues depending on age, hydration, and enamel thickness. A positive Δb* suggests a material appears more yellow than the reference, while a negative Δb* indicates a bluer or cooler tone. Because the human eye is relatively sensitive to changes in yellow-blue contrast, especially under daylight conditions, noticeable differences in b* can compromise the visual integration of restorations [48,49,50]. The results of two-way ANOVA showed that Δb* values were affected by the cement shade and application factors (*p* < 0.05). The results showed that the b* values of the cement shade groups decreased after cementation, except for the opaque cement shade. The b* values were also reduced after thermocycling for all cement shade groups. Furthermore, the difference in b* values between the universal-A2 and clear cement shade groups was significantly higher than the white and opaque cement shades (*p* < 0.05). The results of this study demonstrated that both the shade of the luting cement and the effects of cementation and thermal aging played a significant role in altering the b* color coordinate of the specimens. Most cement shades resulted in a reduction in yellow chroma following cementation and thermocycling, indicating a shift toward a less yellow (or slightly bluer) appearance. This effect was particularly pronounced in the universal-A2 and clear cement groups, which exhibited higher Δb* values compared to the white and opaque cements, suggesting that more translucent or lightly colored cements were more susceptible to chromatic changes over time.

In the present study, the color difference values of the experimental groups were calculated in two stages, after cementation and thermocycling. However, ANOVA results showed that the application (cementation or aging) did not affect the color difference values. Although cementation and aging did not yield statistically significant effects, comparison of color differences with PT and AT thresholds indicated clinically relevant differences. In all groups, cementation and subsequent aging exceeded the clinically acceptable color difference limits (>1.8), except for the opaque cement shade group, which was subjected to thermocycling and remained within the AT threshold.

In the literature, the effects of resin cement color and thermocycling on the color stability of various restoration materials have been investigated. Yamalı et al. [51] investigated the effects of resin cement color, cement thickness, and thermocycling on the final color of monolithic lithium disilicate crowns. Ninety CAD-CAM-milled crowns were cemented on typodont incisors using three cement shades (clear, yellow, and white) and three cement thicknesses (40 µm, 80 µm, and 120 µm). Color parameters were measured before cementation, after cementation, and after 10,000 thermocycles. It was concluded that cementing the restorations with clear, yellow, or white resin cements caused noticeable color differences, except for the group cemented with clear cement at a 40 μm thickness. Following thermocycling, all groups exhibited perceptible color differences. Similarly to Yamalı et al. [51], the present study also highlights that the shade of resin cement and thermal aging can influence the final color of restorations. Although the ANOVA results of the present study did not detect a statistically significant effect of thermocycling, clinically perceptible color changes were observed. Clinical outcomes can differ even when statistical significance is not reached. Furthermore, the difference in color change values between the two studies may be attributed to the variations in the tested restorative materials and study designs. Taşın and Ismatullaev [43] investigated the effect of coffee thermocycling on the color and translucency of five definitive restoration materials, including two 3D-printed permanent crown resins (Formlabs Permanent Crown and VarseoSmile Crown Plus) and three milled materials (lithium disilicate: LDS, polymer-infiltrated ceramic network: PICN, and resin nanoceramic: RNC). Square-shaped specimens (12 × 12 mm) were prepared in 1 mm thickness, and the optical parameters were evaluated before and after 10,000 coffee thermocycles using the CIEDE2000 color difference (ΔE_00_) and RTP_00_ translucency parameters. It was concluded that all tested materials exhibited a decrease in lightness and translucency, as well as an increase in redness and yellowness, after undergoing coffee thermocycling. Among the materials, VSP and PC exhibited the highest color change, while LDS showed the least. Although all materials became darker, more yellow, and less translucent, the overall changes remained within clinically acceptable limits. However, direct comparisons cannot be made with the present study, as no cementation procedure was used, and the tested 3D-printed resins were different. Bozoğulları and Temizci [52] evaluated subtractive (Cerasmart 270 and Vita Enamic) and additive (Crowntec and Permanent Crown Resin) manufacturing techniques, along with a conventional feldspathic glass ceramic (Vita Mark II), to compare their color stability, stainability, and surface roughness. All materials maintained surface roughness values below the plaque accumulation threshold after thermocycling. However, color changes differed according to the material and staining conditions. Crowntec showed the greatest discoloration after thermocycling and immersion in coffee, while Vita Enamic also exhibited an unacceptable color change in coffee. Cerasmart 270 demonstrated the best resistance to staining, and Vita Mark II presented the most stable color performance overall. The findings indicated that both the manufacturing technique and the material composition played key roles in determining the optical and surface properties of restorative materials. Karaoğlanoğlu et al. [53] compared resin-based CAD-CAM blocks (Cerasmart 270 and Grandio Blocs) and 3D-printed permanent resins (Crowntec and Permanent Crown) in terms of surface roughness, microhardness, and color stability after immersion in tea, coffee, and water. Surface roughness remained similar across all groups; however, CAD-CAM blocks exhibited higher microhardness than 3D-printed resins. While initial discoloration was comparable, 3D-printed resins exhibited more pronounced staining after 30 days. 3D-printed resins had lower hardness and higher susceptibility to staining compared to CAD-CAM blocks. Perez et al. [11] evaluated the effect of aging on the color of four 3D-printed resins manufactured with DLP (Detax Freeprint Temp and GC TempPrint) and SLA (Formlabs Temporary CB and Formlabs Permanent Crown) technologies. Specimens were produced in different shades and printing angles, and subjected to accelerated artificial aging. Results showed that all materials experienced clinically unacceptable color changes (ΔE_00_ > 1.8) after aging, with Formlabs Temporary-medium shade and Formlabs Permanent-medium shade showing the least change. Significant alterations in spectral reflectance, transmittance, scattering, absorption, and other optical parameters were observed, regardless of print orientation. The results of these studies highlighted that aging affected the optical stability of 3D-printed dental resins, resulting in a poor color match. These findings suggest that the choice of cement shade not only influences the color outcome but also affects the long-term color stability of the restoration, as thermal aging can modify the optical properties of both the cement and the underlying restorative material.

In the present study, the statistical results showed that only the cement shade had a statistically significant effect on the color difference values. The comparative results showed that the color difference values were significantly higher for the universal-A2 (3.13 ± 1.13) and clear cements (3.08 ± 0.42) than the white (2.10 ± 0.41) and opaque cements (2.09 ± 0.46) (*p* < 0.05). These results suggested that although the 3D-printed resin used in the study was manufactured by Formlabs in A2 shade, its actual color shade appeared to be closer to white or more opaque shades. This discrepancy indicated that the visual characteristics of the material may not fully align with the expected A2 shade standard, which could have implications for esthetic outcomes in clinical applications. Such findings emphasized the importance of verifying the shade accuracy of 3D-printed materials. Furthermore, the color difference values were higher than the clinically acceptable threshold (1.8) for all cement shade groups. This could also be explained by the fact that the 1 mm-thick Formlabs Permanent Resin specimens were translucent enough to reflect the color of the underlying cement. Singh et al. [10] investigated how cement shade and 3D-printing parameters influence the optical properties of additively manufactured (AM) permanent crown materials. Two AM materials (Bego VarseoSmile Crown Plus and Formlabs Permanent Crown) and one milled material (Ivotion PMMA) were tested at 1 mm and 2 mm thicknesses, with AM specimens printed at 0°, 45°, and 90° orientations and cemented using four shades (light, neutral, warm, translucent). Results showed that the neutral cement shade caused the least overall color difference (ΔE_ab_), while translucent cement resulted in the most minor differences between materials. Milled material exhibited higher color changes than AM materials, and material thickness significantly influenced ΔE_ab_ values. Notably, specimens printed at a 45° orientation with 2 mm thickness showed the most clinically acceptable color differences. The findings highlighted that cement shade and printing orientation significantly affected the esthetic outcomes of AM crowns, emphasizing the need for clinicians to carefully consider these factors when selecting materials and procedures for permanent 3D-printed crowns.

Günal-Abduljalil and Ulusoy [46] examined how resin cement shade (A1, A3O, B05, TR), ceramic type (Vita Enamic, GC Cerasmart, and Lava Ultimate), and thickness (0.5 mm and 1.0 mm) affected the final color of resin-ceramic restorations. All factors significantly influenced color outcomes (*p* < 0.05). The A3O cement caused clinically unacceptable discoloration, especially at 0.5 mm, while A1 and TR produced acceptable results, and B05 showed the lowest ΔE_00_ values [46]. In the present study, since the optical properties of thin restorations can be significantly influenced by the cement shade and the aging process, the specimens were prepared with a thickness of 1 mm, which is the recommended minimum restoration thickness for the Formlabs Permanent Crown Resin, as specified by the manufacturer. However, direct comparisons cannot be made between CAD-CAM resin-ceramic materials and 3D-printed resins. CAD-CAM-produced resin-ceramic materials have differing ceramic and resin compositions from 3D-printed resins. In CAD-CAM-produced resin-matrix ceramics, the proportion of resin content is lower compared to 3D-printed permanent composite resins. Formlabs Permanent Crown Resin is composed of 50–75 wt% resin matrix (primarily Bis-EMA) and 30–50 wt% silanized dental glass fillers [17]. These differences in ceramic and resin ratios influence their mechanical and optical properties as well as their clinical indications.

Although the statistical results did not show a significant difference among the groups regarding the effect of cementation and thermal cycling, the mean color difference values indicated that cementation and thermal cycling caused a visually perceptible color alteration in the tested 3D-printed resin. Therefore, it should be recognized that the tested 3D-printed resin may exhibit changes in its optical properties after cementation and following one year of intraoral use. The study also revealed an important clinical finding: the labeled shade of the tested 3D-printed resin (A2) did not correspond to its actual optical appearance, which was closer to white or opaque shades. The results also provide a valuable basis for future research aimed at developing standardized shade-matching protocols, improving the color stability of 3D-printed materials, and conducting long-term clinical studies to assess their esthetic performance over time. However, this study also presents certain limitations. Only one type of 3D-printed photopolymer resin (Formlabs Permanent Crown Resin) was tested, so the findings may not be applicable to other brands or different SLA/DLP resin formulations, which limits the generalizability of the results. Specimens were prepared at a single thickness (1 mm), whereas color differences and translucency can vary with different thicknesses and anatomical crown shapes. Only four resin cement shades were tested, and other commercial cements may exhibit different color behaviors. The study was conducted under laboratory conditions, and thermocycling may not fully replicate the oral environment.

## 5. Conclusions

Both cement shade and cementation/thermocycling had significant effects on the color parameters (ΔL*, Δa*, and Δb*) of the tested SLA-printed photopolymer resin. Opaque cement showed increases in L* and b* values, unlike the other shades. Universal-A2 and clear cements had more stable a* values, while white and opaque cements showed greater shifts.

Cement shade significantly influenced ΔE_00_, with universal-A2 and clear cements showing higher values. As all ΔE_00_ values exceeded the clinically acceptable limit, careful shade selection is essential for the tested 3D-printed resin.

## Figures and Tables

**Table 1:** Detailed information on the permanent crown resin.

Material	Printing Technology	Manufacturer	Printing Parameters	Composition	Applications
Permanent Crown Resin	Stereolithography (SLA)	Formlabs, Somerville, MA, USA	Layer thickness: 50 μm Printing orientation: 90°	Organic Matrix: 50–<75% wt. Bis-EMA Esterification products of 4.4′-isopropylidiphenol, ethoxylated, and 2-methylprop-2enoic acid. Silanized dental glass, methyl benzoylformate, diphenyl [2,4,6-trimethylbenzoyl] phosphine oxide. Inorganic Filler: Silanized dental glass (particle size 0.7 μm) (30–50% wt.)	Permanent single crowns inlays onlays veneers

**Table 2:** The mean values, standard deviations, and comparisons for the ΔL* values.

Cement Shade	Application	
	Cementation (ΔL*-1) Mean (±SD) *n* = 10	Thermocycling (ΔL*-2) Mean (±SD) *n* = 10
**universal-A2**	−3.61 (±1.67) C, b	−1.92 (±1.61) B, a
**clear**	−3.97 (±0.55) C, b	−1.21 (±0.76) B, a
**white**	−2.50 (±0.79) B, b	1.00 (±0.39) A, a
**opaque**	1.43 (±0.83) A, a	1.30 (±0.81) A, a

SD: Standard Deviation. The same uppercase letters indicate that the ΔL* values were not significantly different among the cement shade groups within the same application group (*p* > 0.05). The *p* values for experimental groups after cementation: universal-A2-clear, *p* = 0.436; universal-A2-white, *p* = 0.017; universal-A2-opaque, *p* = 0.0; clear-white, *p* = 0.002, clear-opaque, *p* = 0.00, white-opaque, *p* = 0.00. The *p* values for experimental groups after thermocycling: universal-A2-clear, *p* = 0.125; universal-A2-white, *p* = 0.00; universal-A2-opaque, *p* = 0.0; clear-white, *p* = 0.00, clear-opaque, *p* = 0.00, white-opaque, *p* = 0.521. The same lowercase letters indicate that the ΔL* values were not significantly different between the application groups within the same cement shade group (*p* > 0.05). The *p* values for experimental groups after thermocycling: universal-A2, *p* = 0.00; clear, *p* = 0.00; white, *p* = 0.00; opaque, *p* = 0.771.

**Table 3:** The mean values, standard deviations, and comparisons for the Δa* values.

Cement Shade	Application	
	Cementation (Δa*-1) Mean (±SD) *n* = 10	Thermocycling (Δa*-2) Mean (±SD) *n* = 10
**universal-A2**	1.20 (±0.86) A, a	−1.36 (±0.66) B, b
**clear**	0.83 (±0.48) A, a	−1.39 (±0.44) B, b
**white**	−1.03 (±0.21) B, b	−0.32 (±0.13) A, a
**opaque**	−1.23 (±0.25) B, b	−0.25 (±0.2) A, a

SD: Standard Deviation. The same uppercase letters indicate that the Δa* values were not significantly different among the cement shade groups within the same application group (*p* > 0.05). The *p* values for experimental groups after cementation: universal-A2-clear, *p* = 0.077; universal-A2-white, *p* = 0.00; universal-A2-opaque, *p* = 0.000; clear-white, *p* = 0.00, clear-opaque, *p* = 0.00, white-opaque, *p* = 0.355. The *p* values for experimental groups after thermocycling: universal-A2-clear, *p* = 0.909; universal-A2-white, *p* = 0.00; universal-A2-opaque, *p* = 0.000; clear-white, *p* = 0.00, clear-opaque, *p* = 0.00, white-opaque, *p* = 0.761. The same lowercase letters indicate that the Δa* values were not significantly different between the application groups within the same cement shade group (*p* > 0.05). The *p* values for experimental groups after thermocycling: universal-A2, *p* = 0.00; clear, *p* = 0.00; white, *p* = 0.001; opaque, *p* = 0.00.

**Table 4:** The mean values, standard deviations, and comparisons for the Δb* values.

Cement Shade	Application	
	Cementation (Δb*-1) Mean (±SD) *n* = 10	Thermocycling (Δb*-2) Mean (±SD) *n* = 10
**universal-A2**	−0.29 (±0.65) B, a	−4.77 (±1.02) C, b
**clear**	−0.52 (±0.74) B, a	−5.03 (±0.37) C, b
**white**	−0.67 (±0.70) B, a	−3.53 (±0.61) B, b
**opaque**	3.09 (±1.06) A, a	−2.83 (±0.39) A, b

SD: Standard Deviation. The same uppercase letters indicate that the Δb* values were not significantly different among the cement shade groups within the same application group (*p* > 0.05). The *p* values for experimental groups after cementation: universal-A2-clear, *p* = 0.488; universal-A2-white, *p* = 0.251; universal-A2-opaque, *p* = 0.000; clear-white, *p* = 0.646, clear-opaque, *p* = 0.00, white-opaque, *p* = 0.00. The *p* values for experimental groups after thermocycling: universal-A2-clear, *p* = 0.416; universal-A2-white, *p* = 0.00; universal-A2-opaque, *p* = 0.000; clear-white, *p* = 0.00, clear-opaque, *p* = 0.00, white-opaque, *p* = 0.037. The same lowercase letters indicate that the Δb* values were not significantly different between the application groups within the same cement shade group (*p* > 0.05). The *p* values for experimental groups after thermocycling: universal-A2, *p* = 0.00; clear, *p* = 0.00; white, *p* = 0.00; opaque, *p* = 0.00.

**Table 5:** The mean values, standard deviations, and comparisons for the ΔE_00_ values.

Cement Shade	Application		Total ΔE_00_
	Cementation (ΔE_00_-1) Mean (±SD) *n* = 10	Thermocycling (ΔE_00_-2) Mean (±SD) *n* = 10	
**universal-A2**	3.02 (±1.41)	3.24 (±0.81)	3.13 (±1.13) A
**clear**	3.19 (±0.49)	2.98 (±0.32)	3.08 (±0.42) A
**white**	2.19 (±0.46)	2.02 (±0.37)	2.10 (±0.41) B
**opaque**	2.42 (±0.40)	1.75 (±0.15)	2.09 (±0.46) B

SD: Standard Deviation. The same uppercase letters indicate that the ΔE_00_ values were not significantly different among the cement shade groups (*p* > 0.05). The *p* values for experimental groups: universal-A2-clear, *p* = 0.966; universal-A2-white, *p* = 0.00; universal-A2-opaque, *p* = 0.00; clear-white, *p* = 0.00, clear-opaque, *p* = 0.00, white-opaque, *p* = 1.

## Data Availability

The original contributions presented in this study are included in the article. Further inquiries can be directed to the corresponding author.

## Abbreviations

The following abbreviations are used in this manuscript:

3D	Three-Dimensional
AM	Additive Manufacturing
CAD-CAM	Computer-Aided Design—Computer-Aided Manufacturing
DLP	Digital Light Processing
LCD	Liquid Crystal Display
SLA	Stereolithography
STL	Standard Tessellation Language
